# Risk premia and the European government bond market: new empirical evidence and some thoughts from the perspective of the life insurance industry

**DOI:** 10.1007/s12297-021-00503-2

**Published:** 2021-09-07

**Authors:** Johannes Tholl, Tobias Basse, Samira Meier, Miguel Rodriguez Gonzalez

**Affiliations:** 1 Universität Bayreuth, Universitätsstraße 30, 95447 Bayreuth, Germany; 2 Norddeutsche Landesbank Girozentrale, Friedrichswall 10, 30159 Hannover, Germany; 3 Touro College Berlin, Am Rupenhorn 5, 14055 Berlin, Germany; 4 Mendel University Brno, Zemědělská 1665, 61300 Brno-sever-Cěrná Pole, Czech Republic; 5 Leibniz University Hannover, Otto-Brenner-Straße 7, 30159 Hannover, Germany

**Keywords:** G12, G18, G22, G28, G52.

## Abstract

We study yield spreads between government bonds in the European Monetary Union. This segment of the global fixed income market is of particular importance for insurance companies in Europe. Our empirical research strategy is inspired by Gunay (2020) who has analyzed the relationship between credit and liquidity risk in the United States using Granger causality tests. More specifically, we employ the procedure developed by Toda and Yamamoto (1995) to test for Granger causality among yield spreads in five different member countries of the European Monetary Union (namely Austria, Belgium, France, Italy and Ireland) relative to Germany. We examine interest rate data from bonds with three different maturities (5, 10 and 30 years). Given the importance of long-term bonds as asset class for European life insurers and pension funds, the empirical results from the often ignored market for government bonds with a maturity of 30 years should be of interest. With regard to long-term sovereign debt, there is no evidence for Granger causality among the time series examined here. Consequently, the risk premia required by investors to hold government bonds of one specific member country of the EMU do not help to forecast the risk premia that have to be paid by other countries. Given the structure of their liabilities, this empirical finding should be of high relevance for portfolio and risk managers in the European life insurance industry and in pension funds. With regard to the yield spreads to be observed in the market for 10-year government bonds, there seems to be no clear picture. Focusing on fixed income securities with a maturity of 5 years, there is one very interesting empirical finding. The test results reported here seem to imply that there is unidirectional Granger causality running from the yield spreads in all other four countries to Austria. Given that Austria is a comparably small country which is assumed to be in a fiscally stable position, this result could be interpreted as evidence for credit risk premia as being helpful to forecast liquidity risk premia in the market for medium-term government bonds issued by member states of the European Monetary Union.

## Abstract (de)

Diese Studie untersucht Zinsdifferenzen am Markt von Staatsanleihen der Mitgliedsländer der Europäischen Währungsunion. Dieses Segment des globalen Rentenmarktes hat eine besondere Bedeutung für europäische Versicherungsunternehmen. Unsere empirische Studie ist von Gunay (2000) inspiriert, der den Zusammenhang zwischen Kredit- und Liquiditätsrisiko in den Vereinigten Staaten mittels Grangerkausalitätstests untersucht. Genauer gesagt findet hier der Ansatz von Toda und Yamamoto (1995) Anwendung. Untersucht werden die Zinsdifferenzen von fünf Ländern (Österreich, Belgien, Frankreich, Italien und Irland) zu Deutschland. Dabei wird auf drei Laufzeiten (5, 10 und 30 Jahre) geblickt. Der häufig in empirischen Studien ignorierte Markt für Staatsanleihen mit einer Restlaufzeit von 30 Jahren dürfte aufgrund der Struktur der Verbindlichkeiten von besonderem Interesse für Lebensversicherer und Pensionsfonds sein. In diesem Segment des europäischen Staatsanleihemarktes konnten wir keine Hinweise auf Grangerkausalität zwischen den Zinsdifferenzen finden. Die von den hier betrachteten Ländern für ihre Schulden zu zahlenden Risikoprämien helfen somit nicht, die Risikoprämien in den jeweils anderen untersuchten Nationen vorherzusagen. Dieses Ergebnis sollte von hoher Bedeutung für Kapitalanleger und Risikomanager bei europäischen Lebensversicherungen und Pensionsfonds sein. Im Laufzeitsegment 10 Jahre ergibt sich kein klares Bild. Bei den Zinsdifferenzen der Papiere mit einer Laufzeit von 5 Jahren zeigt sich dagegen klar, dass die Risikoprämien in allen anderen Ländern helfen, die Zinsdifferenz von Österreich zu Deutschland vorherzusagen. Da Österreich eher ein kleines Land mit relativ soliden Staatsfinanzen ist, mag dieses Ergebnis ein Hinweis darauf sein, dass das Kreditrisiko in diesem Segment des europäischen Rentenmarktes zur Prognose des Liquiditätsrisikos verwendet werden kann.

## Introduction

Low interest rates are currently a major problem for the European life insurance industry (see, for example, Basse et al. 2014 and Berdin and Gründl 2015). As a matter of fact, Berdin and Gründl (2015) have argued convincingly that prolonged periods with low long-term interest rates can be regarded as a possibly very dangerous threat to the solvency of those life insurers in Europe that, in the past, have extensively sold policies with expensive guarantees to their customers. This problem is particularly acute in the case of those life insurers that have invested in fixed income securities with durations shorter than those of their liabilities. In any case, the current interest rate environment has caused a hunt for yield among investors that traditionally prefer to buy high quality fixed income securities (see, for example, Conner 2016 and Boubaker et al. 2017). Generally speaking, the low level of interest rates observed today regarding low-risk bonds denominated in Euro is, of course, a direct consequence of the European Central Bank’s (ECB) monetary policy. This policy has applied conventional and unconventional tools to provide stimuli to the crisis-shaken economies in the currency union (see, for example, Burriel and Galesi 2018 and Rodriguez Gonzalez et al. 2019). As will be discussed subsequently in more detail, the severe fiscal problems faced by some countries that belong to the European Monetary Union (EMU) have also caused fears about sovereign credit risk and redenomination risk among investors. As a consequence, risk premia have increased resulting in higher yield spreads of bonds issued by countries that suffer from fiscal challenges. In fact, given the regulatory environment (Solvency II) implemented in the European Union (EU) it could be an interesting option for life insurers to buy government bonds issued by member states of the EMU that have to cope with budgetary difficulties (see, most importantly, Basse et al. 2012 and Ludwig 2014).

The rather high risk premia, that the EMU member countries with fiscal imbalances have to pay in order to issue bonds at the moment, certainly could help life insurers to cope with the problems originating from the guarantees embedded in the old policies they have sold to their customers. However, as Lempérière et al. (2017) have persuasively outlined, there are still major problems when trying to explain how risk premia are determined. Additional empirical evidence with regard to interest rate differentials between government bond yields issued by EMU member countries, with and without budgetary problems, certainly is of importance. Currently, the literature examining sovereign yield spreads in the Eurozone seems to follow a macroeconomic approach by, for instance, analyzing the role of the volume of government debt relative to the respective real gross domestic product or the terms of trade as explanatory variables for interest rate differentials (see, amongst others, Maltritz 2012 and Oliveira et al. 2012). This paper takes a different approach by focusing on the information flow between the sovereign yield spreads, examining data from selected member countries of the EMU. To be more precise, lead-lag relationships between interest rate differentials in a number of member countries of the common currency area are examined in detail. Consequently, the question of predictability is another issue. In other words, it is analyzed whether specific interest rate differentials can help to predict other yield spreads. In order to do so, the concept of Granger causality is employed (see, most importantly, Granger 1969). More specifically, the procedure suggested by Toda and Yamamoto (1995) is used to test for Granger causality (respectively Granger non-causality). Gunay (2020) has already applied this technique to analyze the relationship between liquidity risk and credit risk in the United States. Our study tries to further explore this issue. As already noted we focus on data from the European government bond market. Moreover, the results of our empirical investigations are then primarily assessed from the perspective of the European life insurance industry. However, these findings obviously should also be of interest for the financial economics community in general.

The paper is structured as follows: Section 2 considers the role of government bonds as asset class for European life insurers. In the 3rd section, regulatory issues are examined focusing on Solvency II. Section 4 then briefly addresses the relevant types of risk. The 5th section discusses the tendencies towards interest rate convergence in the currency union after the introduction of the Euro, and then considers the role of the European sovereign debt crisis as well as other related problems. In this context, the ECB’s monetary policy response to the economic crisis caused by the on-going Covid-19 pandemic in Europe and other parts of the world is considered in the 6th section. After discussing some relevant methodological issues, the data is presented in the 7th section. The results of our empirical investigations are discussed and evaluated in section 8. The last section then concludes.

## Government bonds as asset class for European life insurers

Since long-maturity sovereign bonds are an asset class of particular importance for long-term investors like life insurance companies, this chapter sheds some light on the manifold reasons for the relevance of this asset class for European life insurers. In general, life insurers’ business models are broadly clustered into two product categories: life risk products covering the risk of mortality, and life savings products covering the risk of longevity. Especially the old-age provision business of life insurance is particularly susceptible to interest rate changes. Because of these liabilities with a high duration, the investment horizon of life insurers is rather long-term oriented. This fact may even help to stabilize financial markets by anti-cyclical investment behavior, respectively stimulating economic growth (see, for example, Della Croce et al. 2011 and Focarelli 2017). This highlights the macroeconomic relevance of this financial sector, even though, in the case of sovereign bonds, there are indications of a pro-cyclical investment behavior in economic crises—like the European sovereign debt crisis (see, for instance, Bijlsma and Vermeulen 2016 and Fache Rousová and Giuzio 2019). Moreover, Düll et al. (2017), find evidence for a transmission of sovereign risk to the default risk of insurance companies in the wake of the European sovereign debt crisis, which further illustrates the usefulness of empirical evidence on the lead-lag relationships of EMU sovereign yield spreads. Obviously, the ability to predict future developments of government bond spreads is not only of interest to risk and asset managers in the life insurance industry, as well as policymakers and regulators, but also to pension funds and other long-term investors with high exposure to sovereign bonds in their portfolios.

As already stated above, investors worldwide faced aggravating developments in capital markets in the follow-up of the Global Financial Crisis. Indeed, Domanski et al. (2017) argue that in case of the EMU, the relevance of long-term government bonds has increased during the European low interest rate environment (see, for instance, ECB 2015 for a detailed discussion of the difficulties faced by the European insurance sector in a prolonged period of low interest rates). Overall, yields on European government bonds have fallen sharply, not only due to the aforementioned hunt for yield among European investors, but also because of a self-reinforcing herding effect and a hunt for duration in the insurance sector, which is to some extent explained by an increasing negative duration gap (see Domanski et al. 2017). Likewise, Gründl et al. (2017) argue, that in the context of sovereign bonds, life insurers are especially interested in long-maturity bonds to match the duration of their assets to their mostly long-term liabilities. According to the 2018 EIOPA insurance stress test report, the average duration of sovereign bond assets is 7.4 years in the insurance industry, in contrast, the average duration of technical provisions (weighted Macaulay) amounts to 12.5 years for life insurers, and thus, indicating an asset liability mismatch (Battiston et al. 2019). Especially large providers of savings products have to deal with a long-term debt structure.

**Fig. 1:**
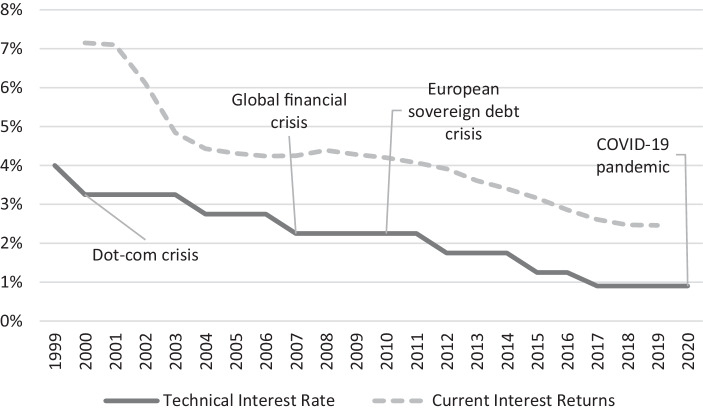
The current interest rate and the technical interest rate of German life insurance companies. (Source: Own representation based on Statista Research Department (2020) and German Association of Actuaries (2020).)

To demonstrate the negative effects in the insurance sector, Fig. 1 shows the guarantee rate contained in classic German life insurance products and the average current interest rate (the sum of the operating profit participation and the guaranteed interest for the life insurance industry weighted by market share) for new business with classic annuity policies. Since the calculation of the maximum technical interest rate is based on average historical government bond yields (see Eling and Holder 2013), the figure shows that both values are continuously decreasing over time, undoubtedly, because of past financial and economic crises and the current low interest rate environment. Since many insurance contracts have a maturity of several decades and some older policies carry interest rates of up to 4%, many life insurers in Germany still have guarantee obligations of around 2–3% in their portfolios. Accordingly, there is a combination of existing high yield liabilities and continuously decreasing average yields in the traditional life insurance business.

In fact, the ECB’s expansive monetary policy reduced interest rates in Europe, which further challenged the EMU insurance market’s returns due to a high sensitivity to interest rate changes in this sector (see, for instance, Van Riet 2017 and Jareño et al. 2020). Berdin and Gründl (2015) state that the impact of the ongoing low interest rate environment will be particularly strong for small and medium-sized life insurance companies that are invested strongly in sovereign bonds. According to the authors, two major features of the life insurance industry trigger these effects: firstly, the high share of fixed income securities in insurers’ portfolios, and secondly, the high sensitivity of interest rate effects on discount rates of insurance liabilities. Besides the current interest rate landscape that puts further pressure on government bond yields, due to low interest rates combined with high financial obligations (see Niedrig 2015), the impact of the Covid-19 pandemic could affect the insurers’ investment behavior, for example because of a lack (and possible worsening) of investment opportunities. However, as described in more detail below, there are many indications that investments in sovereign bonds will prevail, as this is still a preferred investment strategy for the European life insurance industry.

In general, according to Fache Rousová and Giuzio (2019), there are at least five aspects that may influence the insurers’ investment behavior: Namely “[…] the type of firm and its business model, the structure of the balance sheet, the investment preferences of its management and stakeholders, market developments and the regulatory framework under which an insurance firm operates.” (see Fache Rousová and Giuzio 2019, p. 8). Furthermore, when compared to property-liability insurance, life insurers are interested in generating stable cash flows to generate a more predictable calculation of payouts in life insurance products. Moreover, in the life insurance business, the policy provider and the policyholder usually have a business relationship lasting many decades. For this reason, customers’ trust in the long-term solvency of the insurance company is of central importance. Therefore, life insurers are known as conservative investors in the institutional environment, as they are primarily interested in secure investments with low volatility (see Focarelli 2017). As a result, the relationship in European (long-term) government bond yield spreads is of special importance for asset managers in the insurance industry.

In the case of the EMU, also tighter regulatory and solvency requirements, put pressure on investment strategies of pension funds and insurance companies (Gründl et al. 2017). Due to the issuing country’s membership in the currency union, EMU government bonds in particular were seen as safe investments—at least until the default of Greece in 2012. In addition, government bonds are particularly important to life insurers because of their regulatory treatment under the Solvency II Directive in EMU countries (see Ludwig 2014 and Braun et al. 2017). In fact, the regulatory minimum capital requirements under the Solvency II regime enable the regulator to provide incentives for supposedly safe asset classes—for example, EMU government bonds. This will be discussed in more detail later on in chapter 3. However, Düll et al. (2017) find empirical evidence for regulatory flaws in the Solvency II Directive related to risks in insurers’ government bond portfolios being crucial drivers of insurers’ default risk in Europe. To clarify, the equal regulatory treatment of government bonds issued by EMU countries in the internal risk model motivates insurers to invest in European sovereign bonds with the same capital backing requirements, but higher risk premia at the same time. Therefore, in our study, compared to “safe haven assets” like German government bonds, we will analyze both, European government bonds with higher risk premia (like Italy) and lower risk premia (like Austria). Other classification categories are core member states (Austria, Belgium, France) and peripheral member states (Ireland) of the EMU.

The importance of sovereign bonds as asset class is also illustrated by current investment data on the asset structure in the European insurance industry. In Europe, the life insurance sector accounts for 53.62% of all insurers’ assets in the second quarter of 2020 and is consequently the largest investor in this industry.^1^ In the first quarter of 2020, these companies invested primarily in fixed income products like bonds (65.66%). Table 1 shows that the largest share of capital is invested in government bonds (33.63%) and corporate bonds (30.02%) followed by investment funds (16.95%). These collective investment undertakings represent additional important channels for investing in fixed income securities (see Fache Rousová and Giuzio 2019). Additionally, Table 2 shows the relative and absolute exposures to government bonds in the portfolios of European insurers for the countries analyzed in this paper. The data shows that a high proportion of exposure arises in the domestic market (except in the case of Ireland), but also a large proportion of the total exposure to government bonds in other EMU countries. In brief, Table 1 shows the importance of EMU government bonds as asset class for European insurers, whereby Table 2 stresses the particular importance of being able to identify cross-country lead-lag movements in EMU government bond yield spreads because of the high exposure of bonds of other EMU member countries. As already discussed, insurance market data confirm the relevance of information on this asset class. The EIOPA data shows that Europe’s insurance industry is mostly invested in government bonds. However, long-term government bonds are of particular importance for European life insurers, as the average duration of assets is highest for them.

**Table 1:** Investment behavior of European insurance companies (in %). (Source: Own representation based on EIOPA (2020a).)

Investments (other than assets held for index-linked and)	2018 Q2	2018 Q3	2018 Q4	2019 Q1	2019 Q2	2019 Q3	2019 Q4	2020 Q1
*Propeirntkye (do tchoenrt trhaacnts for own use)*	3.19	3.27	3.42	3.27	3.22	3.04	3.11	3.31
*Holdings in related undertakings, including participations*	6.35	6.25	6.37	6.55	6.13	5.04	6.45	6.08
*Equities*	5.04	5.05	4.08	4.29	4.23	3.85	3.76	3.72
Equities—listed	4.15	4.12	3.14	3.37	3.33	2.89	2.90	2.69
Equities—unlisted	0.89	0.93	0.95	0.92	0.90	0.96	0.86	1.03
*Bonds*	69.65	69.59	67.32	66.46	66.34	66.37	64.82	65.66
Government Bonds	34.95	34.78	33.85	33.29	33.37	34.26	32.51	33.63
Corporate Bonds	32.48	32.63	31.42	31.09	30.95	30.18	30.46	30.02
Structured notes	1.39	1.36	1.27	1.23	1.21	1.17	1.31	1.18
Collateralised securities	0.84	0.82	0.77	0.85	0.81	0.77	0.54	0.84
*Collective Investments Undertakings*	13.40	13.57	16.40	16.56	17.04	17.90	18.45	16.95
*Derivatives*	1.21	1.14	1.30	1.64	1.88	2.67	2.27	3.09
*Deposits other than cash equivalents*	0.83	0.82	0.78	0.90	0.82	0.77	0.77	0.86
*Other investments*	0.32	0.31	0.34	0.32	0.33	0.34	0.36	0.34

**Table 2:** Insurance companies’ asset exposure of CIC 1 government bond assets in selected European countries in Q2 2020. (Source: Own representation based on EIOPA (2020b).)

Country	Exposure	Austria	Belgium	France	Germany	Ireland	Italy	Total
Austria	in %	23.85	9.19	9.92	7.13	3.44	2.95	100
	in EURm	5759	2219	2395	1721	830	711	24,148
Belgium	in %	4.09	52.69	13.16	5.05	1.91	4.57	100
	in EURm	5821	75,056	18,745	7190	2714	6511	142,440
France	in %	2.60	5.42	65.08	2.92	1.08	5.52	100
	in EURm	19,638	40,880	490,854	22,053	8142	41,670	754,270
Germany	in %	4.67	7.78	9.48	41.21	1.77	0.94	100
	in EURm	18,015	30,027	36,589	159,096	6816	3639	386,101
Ireland	in %	4.01	3.67	19.31	13.89	6.43	9.49	100
	in EURm	1732	1586	8345	6002	2778	4102	43,224
ITALY	in %	23.85	9.19	9.92	7.13	3.44	2.95	100
	in EURm	2060	6127	13,742	5719	3780	330,822	415,895

To conclude, life insurance companies and pension funds are long-term investors and, therefore, of particular importance for the financial and economic development. Besides, negative impacts on capital investments of institutional investors are likely to endure, for example, due to the economic impact of the Covid-19 pandemic. Moreover, new risks in insurers’ sovereign bond portfolios could emerge—like, for example, climate risks (see, for example, Battiston et al. 2019). However, it can be expected that insurers will continue to be increasingly invested in government bonds in the future. If the exposure is even increased, for example to lower the negative duration gap, a higher share of long-term fixed income securities would also imply higher risks of interest rate changes in the insurers’ portfolios. Such developments could further aggravate the already precarious situation to a so-called “double blow”, as for example happened in Japan in the 1990s. Because of various risk scenarios, like a long-lasting low interest rate environment, as well as the danger of a “double blow”, or the danger of rising interest rates, our empirical investigation is of specific interest for the insurance industry. Therefore, empirical evidence on the information flow among sovereign yield spreads could be helpful for improving financial risk measures in insurers’ asset liability management approaches.

## Some regulatory issues

Aiming to harmonize the EU’s regulatory landscape, a reform process targeting the European insurance industry was introduced resulting in a renewed and modernized regulatory framework—the Solvency II Directive (2009/138/EC) (see, for example, Doff 2008 and Ashby 2011). The establishment of a universal industry standard and the underlying political process are widely regarded as ambitious (see, for instance, Smith 2010 and Basse 2020). Amongst others, Quaglia (2011) and Van Hulle (2011), provide an overview of this political reform process and the underlying drivers. Despite its approval in 2009, the Solvency II Directive only entered into force in 2016. Delays and amendments (for example, the Omnibus II Directive approved by the EU Parliament in 2014), which may at least be partly attributed to the emergence of the sovereign debt crisis, prolonged the process (see, most importantly, Doff 2016). In addition to harmonizing the EU insurance market and improving EU insurers’ competitiveness, Solvency II mainly aims at promoting a more resilient regulation, effective risk management and transparency (see, for instance, Rae et al. 2018 and Hopt 2013).

To achieve the latter, a so-called three-pillar structure had been designed: the first of the three pillars established quantitative regulation of insurance companies’ capital requirements, e.g. the market-consistent valuation of assets and liabilities as well as the determination of the minimum capital requirements (see, for example, Liebwein 2006 and Braun et al. 2018). Hereby, capital requirements for insurance companies in the European Union are harmonized and quantitative reporting is imposed. The second pillar contains qualitative elements of supervision, such as principles for internal risk management and control as well as the supervision of such (see, most importantly, Elderfield 2009). The third pillar predominantly concerns transparency and disclosure requirements, for example, provision of data and information to the supervisor with the overarching aim to promote market discipline (see, for example, Eling et al. 2007 and Liebwein 2006). The three-pillar structure follows a twofold objective: on the one hand, policy holders shall be protected as insurers are required to hold sufficient economic capital, and on the other hand, financial stability is increased (see, amongst others, Boonen 2017 and Gatzert and Wesker 2012). Besides its complexity (see, for instance, Monkiewicz 2013 and Meier, Rodriguez Gonzalez and Kunze, 2020), the Solvency II Directive and its risk-based approach is regarded as highly sophisticated and viewed as a significant improvement to previous regulatory frameworks governing the EU’s insurance industry (see, for example, Rae et al. 2018 and Doff 2016).

However, Solvency II does not come without criticism. For example, Eling et al. (2007) review the cost appropriateness of Solvency II, whereas Monkiewicz (2013) criticizes comprehensiveness and complexity which could be viewed as indicators of compliance costs insurers face. Moreover, another crucial area with room for improvement is addressed in this paper, namely sovereign credit risk under Solvency II.

Vis-a-vis, it is investigated how sovereign credit risk is treated under the three pillars of Solvency II. This evaluation shall help determine whether the current regulatory framework adequately reflects this specific type of risk. With respect to Pillar I, the solvency capital requirements (SCR) specify the amount of funds insurers shall constantly hold in order to withstand an extreme crisis with significant losses. This is a formula-based figure which is newly determined every 12 months quantifying various risks and intending to ensure that insurance companies may avoid default with a 99.5% probability (see, most importantly, European Parliament 2009). In essence, there are two possible approaches to calculate the SCR: (1) applying an internal, bespoke model which requires approval by the supervisor or (2) using the so-called European standard formula (see European Parliament 2009). When applying the standard formula, however, sovereign bonds issued by member states of the European Economic Area (EEA) are classified as risk free with zero risk weight (this has already been discussed briefly in section 2—moreover see, for instance, Basse et al. 2012 and Ludwig 2014). In other words, when an insurance company’s regulatory capital requirements are calculated with the standard formula, sovereign credit and default risks are neglected. As a result, these risks are not accounted for under Pillar I of the Solvency II Directive when quantitative risk-based calculations of capital are conducted from a regulatory point of view.

Simultaneously, it should be noted, that Pillar II of the governance system requires insurers to thoroughly examine their sovereign risk exposure. To be precise, under Pillar II insurers are supposed to undertake the so-called own risk and solvency assessment (ORSA), a strategic analysis of an individual company’s risk profile and risk management practice to be published as a qualitative report (see, amongst others, Düll et al. 2017; European Parliament 2009). The ORSA aims to ensure that solvency needs related to an individual insurer’s risk profile are met, particularly those that are not included or only partly included in the risk assessment based on the standard formula. Consequently, as European government bonds have a zero-risk weight under the standard formula, sovereign risk is supposed to be one of the relevant factors to be determined in the ORSA. In theory, insurers exposed to significant sovereign risk shall reflect scenarios like default of one or more states in their stress tests (Von Saldern 2016). However, ORSA remains ill-defined, especially with respect to the interplay with the calculation of the aforementioned capital requirements (see, most importantly, Gründl and Gal 2013). Ergo, in practice, the results and analysis presented in ORSA reports are not always reliable; this has, for example been stressed by Grima (2017).

Additionally, Pillar II is based on the so-called prudent person principle which states that insurers are only allowed to invest in those kinds of assets of which they are able to properly assess, measure, monitor and manage risks (see, most importantly, European Parliament 2009). Naturally, this also applies to sovereign bonds (Von Saldern 2016). Moreover, as outlined in Art. 5 (1) of the amendment to the Credit Rating Regulation of 2013, insurers are required to undertake their individual credit risk assessments, including risk assessment of government bonds or any other financial instrument contained in their portfolios (see, for example, European Parliament 2013 Von Saldern 2016). For example, indicators like political stability, quality of governance (see, most importantly, Boysen-Hogrefe 2017) as well as a comparison of national economic indicators, such as budget deficits or debt-to-GDP, are useful to properly assess a sovereign bond’s default risk (see, most importantly, Maltritz and Molchanov 2014).

Considering the aforementioned challenges and the long-term low interest rate environment in particular, it has become crucial to review the sovereign credit risk treatment under Solvency II, specifically under the standard formula. Due to the zero-risk weight under the standard formula, any government bond that is issued by any EEA member state in its domestic currency is exempt from solvency capital requirements (see, for instance, Basse et al. 2012 and Ludwig 2014). In consequence, Solvency II does not account for sovereign default risk and ignores sovereign credit risk differentials of member states. Thus, from a regulatory point of view, government bonds issued by countries with comparably larger fiscal imbalances, like e.g. Italy or Spain, are viewed as equally risky and equally unlikely to default as those sovereign bonds issued by fiscally stronger member states, such as Germany, Austria or Finland (see, for example, Basse et al. 2012 and Basse 2020). However, this approach is problematic as government bonds are exposed to individual credit and default risks (see, most importantly, Chaumont 2020). In fact, this has been particularly demonstrated during the Sovereign Debt Crisis in the European Monetary Union (see, most importantly, Meier, Rodriguez Gonzalez and Kunze, 2020). Still, due to the classification as risk-free under SCR, these specific risks are neglected (see, for instance, Basse et al. 2012 and Ludwig 2014). Yet, empirical evidence further proves that sovereign credit risk is priced in by market participants in government bond markets (see, amongst others, Bernoth et al. 2012 and Gruppe and Lange 2014). As pointed out by Basse et al. (2012), it is important to note that regulatory arbitrage may arise when sovereign credit risk is disregarded under Solvency II as this specific risk is generally feared by at least some financial market participants (see, for example, Gruppe and Lange 2014 and Ludwig 2014).

## Risk premia and different types of risk

Risk premia in the segments of the fixed income market that are examined in this paper mainly seem to be driven by three different types of risk—namely liquidity risk, sovereign credit risk and redenomination risk. While liquidity certainly is a key concept in financial economics, there seems to be no well-accepted definition for this important type of risk. Most observers would probably accept the idea that liquidity risk is the risk that a specific asset cannot always be sold without causing a price drop due to a lack of demand for this particular asset. Boudoukh and Whitelaw (1993) have stressed the fact that the value of liquidity seems to be the result of uncertainty concerning future trading needs of current investors. Investors, for example, might be hit by liquidity shocks that would force them to sell assets at specific points in time when prices may be low (see, for instance, Goldreich et al. 2005 and Officer 2007). In these situations, prices of illiquid assets tend to decline more strongly than prices of more liquid assets. As a consequence, investors should be compensated for the existence of liquidity risk. Phrased somewhat differently, a liquidity risk premium ought to exist. However, buy-and-hold investors normally do not plan to sell assets. Therefore, it might be attractive for these investors to prefer holding illiquid assets (“liquidity premium harvesting”). It could be argued that, due to their business model, life insurance companies—which are characterized by a long-term perspective—might not have problems buying assets that cannot be sold instantly without losses due to their illiquidity (see, for example, Möhlmann 2021 and Chodorow-Reich et al. 2021). Liquidity risk obviously does matter for European government bond prices and is directly related to market size (see, for example, Jankowitsch et al. 2006 and Gómez-Puig 2006). Generally speaking, while other factors are also of relevance (for instance active trading in futures), a larger volume of outstanding government debt ought to increase liquidity. Therefore, the smaller member countries of the EMU (e.g., Finland, Ireland or Portugal) should in principle have to pay higher risk premia than the bigger ones (Germany, France and Italy). In fact, empirically evidence seems to clearly point in this direction (see for example, Jankowitsch et al. 2006 and Gómez-Puig 2006).

As discussed below in more detail, sovereign credit risk and redenomination risk did not seem to matter that much for the pricing of government bonds issued by member states of the currency union in the early days of the Euro (see, for example, Gibson et al. 2014 and Basse, Wegener and Kunze, 2018). This has definitely changed since severe fiscal problems have emerged in some member countries of the EMU in the aftermath of the house price collapse in the United States. In any case, the term sovereign credit risk describes the risk that, because of different possible reasons, governments are unable (for example, due to fiscal problems) or unwilling (for instance, because of certain political pressures) to repay their debt (see, for example, Dincecco 2009 and Rodriguez Gonzalez et al. 2019). Should markets anticipate sovereign defaults, investors certainly will demand a compensation for this risk. Countries that are considered to be vulnerable in this context are therefore likely to have to pay higher interest rates to their investors in order to compensate investors for this risk.

Redenomination risk is a very special type of currency risk (see, for example, Grund 2017 and Rodriguez-Gonzalez et al. 2017). A member state that is leaving a currency union because of, for example, fiscal problems or a very strong currency that is hurting the international competitiveness of the respective state’s domestic economy, could decide to introduce a new currency and to redenominate its outstanding government bonds that are not governed by foreign law (see, for example, Grund 2017 and Lapavitsas 2018). This measure of economic policy would most certainly affect investors that hold these fixed income securities in a negative way because the new currency of the country leaving the monetary union would likely devalue against the currency that is still used by the states that remain in the currency union. Consequently, investors should demand a compensation for holding bonds that could be redenominated in a weaker new currency. As a result, countries would have to offer higher interest rates in order to sell such fixed income securities.

The different types of risk discussed here seem to be interconnected. As a matter of fact, Paltalidis et al. (2015) have argued convincingly that macroeconomic shocks can have effects on the level of liquidity in financial markets. Negative news flow or losses at certain banks may, for example, lead to contagious fire sales of banks. This could have an impact on liquidity in financial markets. In this context, Paltalidis et al. (2015) have highlighted the importance of sovereign credit risk. From this perspective, our empirical research approach to search for lead-lag-relationships among risk premia certainly makes a lot of sense. As already noted, this approach has already been used by Gunay (2020) to examine the relationship between credit and liquidity risk in the United States.

## Interest rate convergence in the European Monetary Union

In January 1999, the Euro became the new currency in initially 11 European countries (see, for example, Pollard 2003 and Gruppe et al. 2017). From this point on, these states have started to form the EMU. The creation of the common currency in Europe resulted in the founding of the ECB, a new supranational institution assuming responsibility for monetary policy in the common currency area (see, for example, Kool 2000 and Pollard 2003). There is only one so-called Main Refinancing Operations Announcement Rate determined by the ECB. This key interest rate is identical in all member states of the monetary union. Consequently, the introduction of the Euro should—more or less by definition—have resulted in a convergence of money market interest rates in the member states of the EMU (see, for example, Holder 1999 and Gruppe et al. 2017). Obviously, the introduction of the Euro not only had substantial impact on money markets, but also on bond markets. In fact, Kim et al. (2006) have argued convincingly that the adoption of the new common currency caused structural change in the European bond market. First of all, short and long-term interest rates are closely connected to each other. Moreover, the Euro has eliminated the influencing factor exchange rate risk for investors situated in one member state buying bonds issued in other countries also belonging to the currency union (see, amongst others, Gómez-Puig 2006 and Gruppe et al. 2017). As a matter of fact, Lund (1999) has argued that even before 1999, there already was interest rate convergence between the bond yields in at least some states that later on introduced the Euro because of the pre-agreed binding timetable and the rules for the adoption of the common currency. In any case, the introduction of the Euro and the founding of the ECB caused strong convergence tendencies among nominal short-, medium- and long-term interest rates in the member states of the EMU.

About one decade later, the European Sovereign Debt Crisis changed the way financial markets priced government debt issued by member countries of the monetary union (see, for example, Gruppe and Lange 2014 and Ludwig 2014). Basse (2014) and Sensoy et al. (2019) have stressed, that during the crisis, there have been two groups of countries—namely those with and those without noteworthy fiscal problems. In the context of this crisis, fixed income investors holding bonds issued by certain member countries of the EMU started to fear sovereign credit and redenomination risk (see, among others, Basse 2014 and Sibbertsen et al. 2014). In this difficult environment, there was no broad convergence of interest rates in the currency union anymore. In fact, even flight-to-quality-effects could be observed back then. The strong demand for German sovereign bonds and those of some other fiscally more stable member states of the currency union pushed down the level of interest rates in these countries (see, for example, Sibbertsen et al. 2014 and Phillips and Shi 2019). Investors indeed seemed to fear a collapse of the financial system in the EMU. As a consequence, the responsible economic policy makers saw an urgent need for action. Afonso et al. (2018), for example, have stressed that the ECB’s monetary policy measures taken in August 2012 with the aim to improve the liquidity situation in financial markets seem to have contributed greatly to the reduction of tensions in the market for European government bonds. In fact, meanwhile many observers believe that Mario Draghis’s now famous speech (“whatever it takes”) has helped to more or less completely eliminate the fears prevalent among investors that the EMU could break up (see, for example, Klose and Weigert 2014 and De Vries and De Haan 2016). Phrased somewhat differently, Draghi’s words most probably have dramatically reduced the risk premia compensating buyers of sovereign bonds issued by fiscally weaker member states (like, for example, Italy or Spain) for redenomination risk. Additionally, not only the speech (which certainly had an impact on market expectations) but also the unusual monetary policy measures taken by ECB after Draghi’s words (quantitative easing) seem to further have lowered risk premia (see, amongst others, Krampf 2016 and Krishnamurthy et al. 2018).

As a result, the European government bond market seems to be characterized by at least three different pricing regimes for fixed income securities issued by sovereign states (namely before the crisis, after the crisis and after Draghi). Yet the matter is perhaps even more complicated. Arghyrou and Kontonikas (2012), for instance, have suggested that the sovereign debt crisis in Europe should be divided into an early and a later phase and that the mounting fiscal problems in Greece could be of some importance in this context. The meltdown of the housing market in the United States and its effect on the global financial system may also be of relevance (see, most importantly, Wegener, Kruse and Basse 2019). Accompanied by a higher level of risk aversion among investors due to the collapsing mortgage market in North America, the fears of costly bank bail-out programs in Europe (see Basse et al. 2012 and Wegener, Kruse and Basse, 2017) could, in fact, help to explain, why “all of sudden” a sovereign debt crisis has disrupted the government bond market in the EMU. Therefore, it seems reasonable to distinguish between an early phase of the crisis that probably was caused by problems in the banking industry and a more fundamental macroeconomic crisis in specific member countries. The empirical evidence that has been presented by Ejsing and Lemke (2011) seems to point in this direction. Accepting this perspective, there could be at least four different relevant pricing regimes for government bonds issued by member states of the monetary union after the introduction of the Euro in 1999 (before the crisis, early crisis, late crisis and after Draghi). Moreover, the political turmoil in Italy after the election in 2018 and the monetary policy response to the economic crisis caused by the Covid-19 virus might also have affected sovereign bond markets in Europe. In any case, meanwhile many observers seem to believe that there certainly was an underpricing of sovereign credit risk in the EMU before 2008 (see, for example, Gibson et al. 2014 and Basse, Wegener and Kunze, 2018) and possibly also an overpricing of redenomination risk and sovereign credit risk after the debt crisis in Greece (see, on the one hand, Gibson et al. 2014 and, on the other hand, the more cautionary comments by Afonso et al. 2020).

Italy represents a suitable example where the two aforementioned crises culminated. The country that is home to the oldest bank in the world was hit not only by a sovereign debt crisis, but also by a financial sector crisis that inflicted harm to each other. Domestic banks suffered from Italy’s sovereign rating downgrades that had a negative impact on default rates which adversely affected banks’ balance sheets as these were exposed to large volumes of Italian sovereign assets. The same mechanism applies to receivables against the sovereign. Simultaneously, Italy’s national budget suffered due to the fact that domestic banks fell into financial distress and required financial support from the government (see Tholl et al. 2020).

## How monetary policy aims at combatting Covid-19

As a lesson learned from the Global Financial Crisis, many governments introduced fiscal measures to tackle a symmetric decline in aggregated demand immediately after the Covid-19 virus began to spread around the world. The fiscal impulse was accompanied by monetary stimuli from central banks following the intention to provide crisis relief more swiftly compared to the 2008 Global Financial Crisis (see Haas and Neely 2020). As this kind of economic shock has been unprecedented in its scale and speed of impact, extensive fiscal and monetary responses have been regarded as proportional to the purpose (see Altig et al. 2020). The monetary authorities repeatedly adjusted their key interest rates due to the pandemic induced supply-and-demand shock (see Botta et al. 2020). As a consequence of the interest rate cuts, the gaps between the key interest rates of major central banks narrowed (see Haas and Neely 2020). In order to understand why the ECB adopted the Pandemic Emergency Purchase Program (PEPP) shortly after Covid-19 began to spread in Europe, the learnings from the sovereign debt crisis in 2011/2012 should be taken into account. Valiante (2011) identified two main drivers of the debt crisis: macroeconomic imbalances and flaws in the institutional organization. In fact, some observers seem to belive that the ECB did not adopt the role as lender of last resort and thereby did not manage to prevent yield spreads of sovereign issuers from the periphery of the EMU to rise. This only changed with the introduction of the Outright Monetary Transactions (OMT) which helped to calm the financial markets (see Filoso et al. 2021).

Prior to the outbreak of Covid-19, the United States experienced interest rate levels that had returned towards some kind of normalization, while the ECB maintained its deposit facility rate (DFR) at record low levels. In September 2019, the DFR was reduced even further to −0.50% (see Aguilar et al. 2020). The ECB also continued with its Asset Purchase Programme (APP) comprising of a volume of € 20 billion and claimed to do so until inflation rates would rise (see Boeckx et al. 2020). The ECB aimed at achieving its inflation rate target of close to 2% by keeping this course of expansive monetary policy (see Asshoff et al. 2020). Since July 2019 and April 2021, the ECB has failed to meet its inflation target. The latter has been defined by its Governing Council in 2003, proclaiming its pursuit of price stability that is given when inflation rates remain “below, but close to, 2% over the medium term (see ECB 2021c).” The clarification “but close to” compared to the definition of 1998 can be interpreted as ECB’s intention to eliminate potential deflationary fears (see Paloviita et al. 2021). In times of very low inflation rates, monetary policymakers have to deal with the challenge of navigating between Scylla and Charybdis by either falling into the deflation trap or the inflation trap (see Brunnermeier 2021). This implies the increased risk in case of an external shock, that expansive monetary policy measures conducted to prevent a deflation trap may provoke an over-shooting, and thus, could cause an inflationary spiral. As a result of its current monetary policy review, the ECB adopted a new inflation target of 2% and is willing to tolerate short periods of inflation rates “moderately above target” (see ECB 2021d). This change in the ECB’s monetary policy strategy may be interpreted as an effort to widen the corridor between the deflation and the inflation trap.

**Fig. 2:**
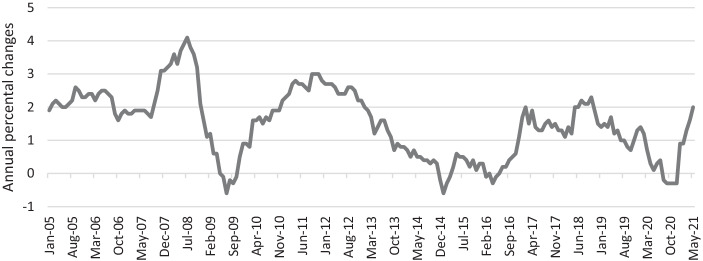
Inflation rate Euro Area (consumer prices). (Source: Own representation based on ECB (2021a).)

With interest rates lowered to levels below zero, traditional monetary tools have limited effect to stimulate economic activity and the ECB continued to adopt unconventional monetary policy instruments as crisis response (see Benmelech and Tzur-Ilan 2020). As a result, the ECB not only expanded but also accelerated its unconventional monetary policy. In consequence, the year 2020 recorded the highest asset purchases per month since the APP was launched (see Fig. 2). The growing ECB balance sheet reflects this process (see Haas and Neely 2020). Thanks to these immediate actions, the central banks’ purchase programmes helped to control the yield curve which is especially beneficial for high-debt countries and corporations that issue investment grade bonds (see Zabala and Prats 2020). Due to the monetizing mechanism, governments suffering from fiscal imbalances are somewhat protected from running into a debt crisis as the central banks’ behavior implicitly guarantees that there is a stable demand for sovereign bonds issued by these countries. This, in turn, keeps interest rates close to those of low-debt countries. Thereby, the ECB aimed at preventing this economic crisis from mutating into yet another sovereign debt crisis (see Blanchard and Pisani-Ferry, 2020). Further support for this argument can be found when considering the announcement of the PEPP program in March 2020, which intended to raise the share of bonds held by ECB by about 30% and helped to narrow yield spreads against German bunds (see Haas and Neely 2020). Particularly Italian sovereign bonds benefitted from the ECB’s extensive monetary stimulus to address the Covid-19 induced economic impact (see Bernoth et al. 2020). This is underlined by Table 3, showing the purchases per country under the PEPP regime. It becomes apparent that the Italian share is significantly disproportionate to its economic importance due to the fact that the country was not only severely hit by Covid-19 but it already suffered from a high debt burden even before the pandemic sparked-off.

**Table 3:** Bimonthly breakdown of public sector securities under PEPP. (Source: Own representation based on ECB (2020a).)

Book value as at end-July 2020 (EUR millions)	Net purchases June-July 2020	Cumulative net purchases as at end-July 2020^b^	Current WAM^a^ of public sector securities holdings under the PEPP^c^	WAM of eligible universe of public sector securities under the PEPP as at end-July 2020^c^
Austria	5,142	10,056	10.01	7.33
Belgium	6,392	12,853	5.83	9.27
Cyprus	455	936	11.79	8.31
Germany	46,266	93,016	3.97	6.60
Estonia	163	163	9.30	7.29
Spain	23,719	46,111	8.18	7.40
Finland	3,225	6,456	7.56	7.07
France	35,845	59,420	9.05	7.07
Greece	5,256	9,946	8.62	9.07
Ireland	2,972	5,972	8.31	9.29
*Italy*	*36,067*	*73,432*	*7.00*	*6.72*
Lithuania	543	1,593	9.21	10.92
Luxembourg	348	807	6.56	5.74
Latvia	391	787	9.88	9.08
Malta	0	123	6.33	8.02
Netherlands	10,285	20,674	3.60	7.36
Portugal	4,655	8,805	7.14	6.81
Slovenia	958	1,896	6.84	8.71
Slovakia	1,487	3,790	7.17	8.13
Supranationals	14,045	27,980	8.23	7.23
Total	198,214	384,817	6.71	7.12

^a^WAM stands for weighted average maturity

^b^Cumulative net purchase figures represent the difference between the acquisition cost of all purchase operations and the redeemed nominal amounts

^c^Remaining WAM in years

Given that the EMU has been shaped by two crucial events—the Global Financial Crisis and the sovereign debt crisis, it may be subject to future discussions, whether ECB’s reaction to the impact of Covid-19 has been a new landmark in the history of the EMU. Since its creation, the EMU faces criticism referring to the theory of optimum-currency area (OCA) which proclaims conditions that should be fulfilled by a common currency area, like providing integrated financial markets, in order to cope with the disadvantages of monetary integration. According to these sceptic views, it is a matter of time that the EMU will collapse in the aftermath of an economic crisis, as the Euro Area does not fully meet the conditions of an OCA. Therefore, the EMU is supposed to lack capacity to cope with severe economic shocks (see Eichengreen 1992). In this regard, the Euro Area proved its resilience during and after the Global Financial Crisis and the following sovereign debt crisis as these events sparked financial fragmentation and put the EMU at risk to break up. The ECB was forced to create instruments to tackle the lack of liquidity in financial markets and later the widening of sovereign bond spreads. Hartmann et al. (2021) have provided evidence that the first weeks of the Covid-19 spreading in Europe also show sharp tendencies of financial disintegration. This was driven by a strong demand for money-market instruments and a widening of sovereign spreads among EMU member states which indicates that the economic impact of the COVID-19 crisis could challenge the stability of the Euro Area like the Financial Crisis and the sovereign debt crisis.

**Fig. 3:**
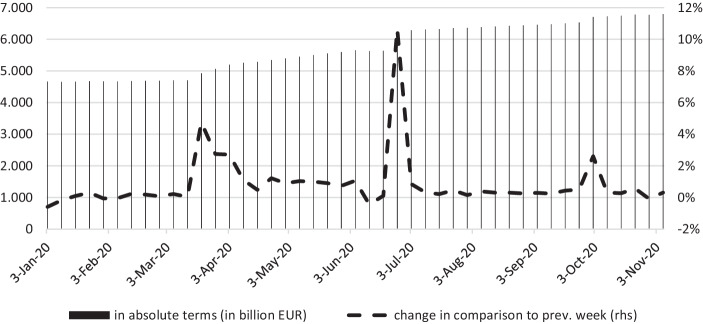
ECB balance sheet. (Source: ECB (2020b).)

In mid-March 2020, the ECB announced the launch of the aforementioned PEPP, including a package of asset purchases and a bank relief program with a volume of originally € 120 billion that was later extended to an amount of € 750 billion, and even further increased to € 1,350 billion in June 2020 (see Fig. 3; Jinjarak et al. 2020), being topped up by further € 500 billion in December 2020 totaling € 1,850 billion (ECB 2021b). Referring to Mario Draghi’s famous “whatever it takes”-quote that helped to calm down market fears (see Claeys 2020), on March 18, 2020 the ECB proclaimed that the PEPP design can be adapted “as much as necessary and for as long as needed” (see Bénassy-Quéré et al. 2020). This program comprises various instruments to not only prevent a credit crunch as consequence of the economic downturn, but also to stabilize markets, so that the monetary policy mechanism is preserved. When investors grasped the economic impact of Covid-19, there was a high risk of liquidity shortfall, and flight to safe-haven assets with potentially severe consequences, especially for highly indebted member states of a monetary union (see Hutchinson and Mee, 2020). Between the beginning of January 2020 and mid of March of the same year, the yield spreads between German government bonds and, for example, Italian sovereign bonds had widened sharply. After the ECB announced its PEPP-program to cushion the economic effects of Covid-19, Italian and Spanish sovereign bond spreads, interrupted by a widening in April, have narrowed (see Boeckx et al. 2020).

Further crisis response by the ECB includes the Governing Council’s decision to extend the additional credit claim (ACC) framework by accepting credit claims as collateral which did not meet the predefined eligibility criteria, inter alia loans with lower credit quality standards (see ECB, 2020c). Furthermore, the threshold for using credit claims as collateral for banks to obtain new liquidity was lowered from formerly € 25,000 to 0. This measure was intended to incentivize an additional credit supply to small and medium enterprises. Another ECB instrument to mitigate the economic impact of the Covid-19 pandemic included an increase in tolerating collateral devaluations by 20% (see ECB 2020d). In order to prevent a liquidity shortfall for the real economy, the ECB aimed at establishing improved refinancing conditions for banks as these play an even more important role as financial intermediary in Europe than in the United States. Hence, the ECB continued to provide targeted (TLTROs) and non-targeted liquidity programmes that intend to ease banks’ borrowing from ECB, a program launched in September 2019 already. Based on the longer-term refinancing operations (LTROs) that have expired in March 2020, the ECB opted for a continuation named pandemic emergency longer-term refinancing operations or short: PELTROs (see ECB 2020e). On the flip side, lending rates for banks turned negative (see Haas and Neely 2020). Another important change includes the ECB’s decision to revise its rules with respect to public bond purchases so that the commitment to hold no more than one third of a country’s outstanding government bond was abolished (see Bernoth et al. 2020).

## Data and methodological issues

This empirical study examines interest rate differentials between 5, 10 and 30 year bond yields in five different member countries of the EMU relative to German sovereign bond yields. Fixed income securities issued by the Federal Republic of Germany are usually considered to be more or less free of default risk. Moreover, there is a very high level of liquidity in the market for German government bonds. Therefore, sovereign bond yields from Germany are frequently used as the benchmark interest rate for the EMU (see, for example, Basse 2014 and Rodriguez Gonzalez et al., 2019). Given the research question under examination here, it is certainly necessary to also consider 30-year interest rate differentials (which is often not done in empirical studies). In fact, Rodriguez Gonzalez et al. (2019) and Basse (2020) have argued convincingly that this segment of the sovereign bond market in the EMU is of special importance for the life insurance industry because of their long-term liabilities. Besides Germany (as benchmark), we examine interest rate data from five other member states of the EMU (namely, Austria, Belgium, France, Italy and Ireland). Austria is a smaller country that in general is assumed to belong to the fiscally more prudent ones. Therefore, liquidity risk should play a special role for bond prices issued by Austria. France and Italy are large member countries of the EMU with highly liquid government bond markets. Consequently, government bond yields in these two countries should not be driven by liquidity risk. Given the aforementioned recent political turmoil in Rome, sovereign credit risk and redenomination risk should indeed be of some importance for Italian government bond prices. Belgium and Ireland are medium-sized respectively smaller member countries of the EMU. In both cases, liquidity risk, sovereign credit risk and redenomination risk could impact government bond prices and interest rates. Moreover, Ireland was among the countries that suffered most during the European sovereign debt crisis (see, for example, Gómez-Puig and Sosvilla-Rivero 2014 and Wegener, Kruse and Basse, 2017).

The yield spread SP of sovereign debt (see, for example, Gómez-Puig 2006 and Rodriguez Gonzalez et al. 2019) issued by country W (Austria, Belgium, France, Italy and Ireland) relative to German bonds with the maturity Z (5, 10 or 30 years) is calculated form generic government bond yields using Eq. 1:

1 $${SP}_{W,Z}=i_{W,Z} - i_{Germany,Z}$$

All interest rate data is taken from Bloomberg. Given that identical maturities are examined and that investors consider German government bonds to be somewhat special—as already discussed, these fixed income securities characterized by high liquidity, and there no fears of a sovereign default—the interest rate differentials computed according to Eq. 1 can be interpreted as risk premia compensating investors for the higher default and liquidity risk of country W relative to Germany (and, of course, also for the possibly existing redenomination risk). We examine weekly data. In order to avoid problems with structural change, the data sample analyzed is 3/29/2019 to 7/03/2020. Focusing on this period of time does make sense because the 10-year German government bond yield was negative for the whole sample. This is a very important fact with regard to the existence of structural breaks in the bond yield spread time series. The procedure suggested by Phillips and Perron (1988) is employed to test for unit roots in the time series calculated with Eq. 1. According to the results of these tests, all yield spreads seem to be non-stationary variables integrated of order 1. Given the empirical findings that have been reported by Rodriguez Gonzalez et al. (2019), this result is not surprising. Therefore, no test data is reported in order to conserve space.

The concept of Granger causality is of high relevance in the field of time series econometrics. One-time series X is Granger causing another time series Y when past values of X can predict the variable Y (see, most importantly, Granger 1969). Expressed somewhat more formally, the variable *X*_*t*_ is said to not be Granger causing the time series *Y*_*t*_ if for all $n> 0$

2 $$F\left(Y_{t+n}\mid \Omega _{t}\right)=F\left(Y_{t+n}\mid \Omega _{t}-X_{t}\right)$$

In Eq. 2, *F* denotes the conditional distribution, and $\Omega _{t}-X_{t}$ is all potentially relevant information except of *X*_*t*_. Feedback effects may exist between the two variables *X*_*t*_ and *Y*_*t*_. Then there is bidirectional Granger causality (see, for example, Thornton 1996 and Amiri and Ventelou 2012). On the other hand, there is unidirectional Granger causality in situations where one variable Granger causes the other variable but not vice versa (see, for example, Oxley 1993 and Thornton 1996).

The Granger causality tests are performed using the approach developed by Toda and Yamamoto (1995). This procedure has become very popular among applied econometricians in recent times. As a matter of fact, Bauer and Maynard (2012) have highlighted how useful this approach to test for Granger causality can be. Due to the large number of relevant studies, we can only give two examples here. Amiri and Ventelou (2012), for instance, have used the technique that has been suggested by Toda and Yamamoto (1995) to examine the relationship between healthcare expenditures and economic activity. Moreover, Kunze et al. (2020) have employed this approach to search for a useful leading indicator of house prices in the United Kingdom. This popularity most probably is a result of the favorable Monte Carlo evidence that has been presented by Zapata and Rambaldi (1997). The technique that has been developed by Toda and Yamamoto (1995) is based on the concept of vector autoregressive models. More specifically, vector autoregressions are very useful tools to describe the dynamic interrelationships between two or more-time series (see, most importantly Sims, 1980). The *n* endogenous variables in a vector autoregressive models are explained by past values of itself and of the remaining other variables examined. In Eq. 3*Y*_*t*_ is a vector of $(n\times 1)$ endogenous variables, *A*_*i*_ are $(n\times n)$ coefficient matrices, *C* is a $(n\times 1)$ vector of constants and *ε*_*t*_ is an $(n\times 1)$ vector of random disturbances:

3 $$Y_{t}=C+A_{1}Y_{t-1}+A_{2}Y_{t-2}+\cdot \cdot \cdot +A_{p}Y_{t-p}+\varepsilon _{t}$$

This technique can account for possibly existing feedback effects among the variables that are included in the model. Toda and Yamamoto (1995) have suggested to estimate a vector autoregression in levels considering *p* time lags and to extend this model by *m* time lags to then perform modified Wald tests to search for Granger causality, where *m* is the highest order of integration of any exogenous variable examined and *p* is the optimal number of time lags for the vector autoregressive model:

4 $$Y_{t}=C+A_{1}Y_{t-1}+A_{2}Y_{t-2}+\cdot \cdot \cdot +A_{p}Y_{t-p}+\cdot \cdot \cdot +A_{p+m}Y_{t-\left(p+m\right)}+\varepsilon _{t}$$

This procedure using a modified Wald test ensures that the test statistic is asymptotically chi-square distributed. The additional m lags in Eq. 4 are added to the augmented model as exogenous variables and *p* is the optimal number of time lags for the vector autoregression that can, for example, be selected by using the traditional information criteria (in or case AIC). Phrased somewhat differently, the null hypothesis of Granger non-causality is tested by only examining the coefficient matrices *A*_1_ to *A*_*p*_. The procedure suggested by Toda and Yamamoto (1995) can be problematic when there is structural change (see, most importantly, Gormus et al. 2018; and Nazlioglu et al. 2019); employing the so-called Fourier Toda Yamamoto test should be helpful in these cases. However, working with small sample sizes (as done here) using the traditional test procedure could have advantages. In fact, Monte Carlo evidence presented by Nazlioglu et al. (2019) does suggest that the test procedure developed by Toda and Yamamoto (1995) seems to be less distorted than the Fourier Toda Yamamoto test examining small samples. Moreover, given that we already have selected the data sample examined here in a way that should help to minimize possible problems with structural change (as discussed above), we prefer to employ the traditional version of the test.

## Empirical analysis

The results of the Granger causality tests (*p*-values) employing the technique suggested by Toda and Yamamoto (1995) are presented in the Table 4 and 5 and 6 and 7 and 8 and 9 and 10 and 11 and 12 and 13. The reported probabilities are calculated using the asymptotic Chi-square distribution. In the tables, X → Y denotes Granger causality running from the variable X to the variable Y, and Y → X denotes Granger causality running from the variable Y to the variable X. Examining the empirical findings that are presented in the tables, there are some very interesting results.

**Table 4:** Granger causality test Austria and Belgium. (Source: Own calculations.)

Maturity	Austria → Belgium	Belgium → Austria
5 Years	0.2973	0.0000
10 Years	0.0199	0.0225
30 Years	0.5081	0.5016

**Table 5:** Granger causality test Austria and France. (Source: Own calculations.)

Maturity	Austria → France	France → Austria
5 Years	0.4560	0.0004
10 Years	0.1272	0.2755
30 Years	0.6941	0.4054

**Table 6:** Granger causality test Austria and Ireland. (Source: Own calculations.)

Maturity	Austria → Ireland	Ireland → Austria
5 Years	0.2130	0.0047
10 Years	0.1151	0.1152
30 Years	0.5237	0.9869

**Table 7:** Granger causality test Austria and Italy. (Source: Own calculations.)

Maturity	Austria → Italy	Italy → Austria
5 Years	0.1522	0.0197
10 Years	0.0741	0.1704
30 Years	0.1343	0.2800

**Table 8:** Granger causality test Belgium and France. (Source: Own calculations.)

Maturity	Belgium → France	France → Belgium
5 Years	0.1223	0.0553
10 Years	0.0066	0.0056
30 Years	0.6603	0.9254

**Table 9:** Granger causality test Belgium and Italy. (Source: Own calculations.)

Maturity	Belgium → Italy	Italy → Belgium
5 Years	0.4027	0.1626
10 Years	0.2156	0.8855
30 Years	0.3214	0.1023

**Table 10:** Granger causality test Belgium and Ireland. (Source: Own calculations.)

Maturity	Belgium → Ireland	Ireland → Belgium
5 Years	0.0686	0.2799
10 Years	0.5297	0.3274
30 Years	0.2591	0.8005

**Table 11:** Granger causality test France and Italy. (Source: Own calculations.)

Maturity	Italy → France	France → Italy
5 Years	0.6450	0.0748
10 Years	0.5462	0.0237
30 Years	0.0905	0.3881

**Table 12:** Granger causality test France and Ireland. (Source: Own calculations.)

Maturity	Ireland → France	France → Ireland
5 Years	0.5007	0.5761
10 Years	0.9434	0.2460
30 Years	0.8838	0.1708

**Table 13:** Granger causality test Italy and Ireland. (Source: Own calculations.)

Maturity	Ireland → Italy	Italy → Ireland
5 Years	0.0455	0.4472
10 Years	0.1477	0.8209
30 Years	0.6689	0.7892

From the perspective of asset managers in life insurance companies, it is of predominant importance to note that with regard to interest rate differentials of bonds with a maturity of 30 years, there is no empirical evidence for Granger causality among the time series examined here. As a matter of fact, in no case the null hypothesis of no causality can be rejected. Consequently, focusing on bonds with high durations yield spreads relative to Germany in one of the member countries of the EMU do not help to forecast yield spreads in the other countries. This is somewhat different in the other segments of the European government bond market. When examining fixed income securities with a maturity of 10 years, there is no clear picture at all. In some cases, there is no Granger causality, in others there is uni- or bidirectional causality. Focusing on medium-term bonds (which here means a maturity of 5 years), there is one very interesting empirical finding. All models do suggest that there exists unidirectional Granger causality running from the yield spreads in all other countries to Austrian interest rate differentials relative to German 5‑year bonds. This result is remarkable. As already noted, Austria is a smaller European country which is considered by most investors to be fiscally very sound. Therefore, yield spreads to Germany mainly are compensating holders of Austrian government bonds for liquidity risk and not for sovereign credit risk. Consequently, the data set examined here seems to suggest that sovereign credit risk, which is reflected by the prices of medium-term European government bonds issued by, for example, Italy or Ireland, can help to forecast liquidity risk premia in this segment of the global fixed income market. One explanation for this empirical finding could be that additional fears about sovereign credit risk can lead to liquidity shocks which then tend to increase liquidity premia. This interpretation of the empirical evidence reported here is, of course, based on the point of view that fixed income investors do not seem to believe that sovereign credit risk per se can become a major problem in Austria.

## Conclusion

Lempérière et al. (2017) have argued convincingly that there still are surprisingly large obstacles when trying to explain how risk premia are determined in financial markets. With this study, we try to close some of the existing knowledge gaps. Doing so, we focus on the government bond market in the EMU. This segment of the global fixed income market is of particular importance for insurance companies in Europe. More specifically, we employ the procedure developed by Toda and Yamamoto (1995) to test for Granger causality among yield spreads in five different member countries of the EMU relative to Germany. The member states included in the analysis are Austria, Belgium, France, Italy and Ireland. We examine interest rate data from bonds with three different maturities (5, 10 and 30 years). Our empirical research approach is inspired by Gunay (2020) who has analyzed the relationship between credit and liquidity risk in the United States using Granger causality tests. With regard to long-term sovereign debt, there is no evidence for Granger causality among the time series examined here. Consequently, the risk premia required by investors to hold government bonds of one specific member country of the monetary union do not help to forecast the risk premia that have to be paid by other countries. Given the structure of their liabilities, this empirical finding should be of relevance for the European life insurance industry. With regard to the yield spreads to be observed in the market for 10-year government bonds, there seems to be no clear picture. Focusing on fixed income securities with a maturity of 5 years, there is one very interesting empirical finding. The test results reported above seem to imply that there is unidirectional Granger causality running from the yield spreads in all other four countries to Austria. Given that Austria is a smaller country which is viewed to be in a fiscally stable position, this result could be interpreted as evidence for credit risk premia being helpful to forecast liquidity risk premia in the market for medium-term government bonds issued by member states of the EMU. Future empirical research that focuses on the European government bond market should examine the relationship between sovereign credit risk and liquidity risk in more detail. Moreover, the empirical research strategy employed here can also be used to improve our understanding of how risk premia are determined in financial markets in general by analyzing lead-lag-relationships between the historical risk premia offered by different types of investment opportunities (e.g., small cap stocks versus growth stocks).

## Footnotes

1 Authors’ own calculations based on EIOPA (2020c).

## References

[CR1] Afonso A, Arghyrou MG, Gadea MD, Kontonikas A (2018). “whatever it takes” to resolve the European sovereign debt crisis? Bond pricing regime switches and monetary policy effects. J. Int. Money Finance.

[CR2] Afonso A, Jalles JT, Kazemi M (2020). The effects of macroeconomic, fiscal and monetary policy announcements on sovereign bond spreads. Int. Rev. Law Econ..

[CR3] Aguilar, P., Arce, Ó., Hurtado, S., Martínez-Martín, J., Nuño, G., Thomas, C.: The ECB monetary policy response to the Covid-19 crisis. Banco España Documentos Ocasionales **2026** (2020)

[CR4] Altig D, Baker S, Barrero JM, Bloom N, Bunn P, Chen S, Davis SJ, Leather J, Meyer B, Mihaylov E, Mizen P, Parker N, Renault T, Smietanka P, Thwaites G (2020). Economic uncertainty before and during the COVID-19 pandemic. J. Public Econ..

[CR5] Amiri A, Ventelou B (2012). Granger causality between total expenditure on health and GDP in OECD: evidence from the toda-yamamoto approach. Econ. Lett..

[CR6] Arghyrou MG, Kontonikas A (2012). The EMU sovereign-debt crisis: fundamentals, expectations and contagion. J. Int. Financial Mark. Inst. Money.

[CR7] Ashby S (2011). Risk management and the global banking crisis: lessons for insurance solvency regulation. Geneva Pap. Risk Insur. Issues Pract..

[CR8] Asshoff S, Belke A, Osowski T (2020). Unconventional monetary policy and inflation expectations in the Euro area. Econ. Model..

[CR9] Basse T (2014). Searching for the EMU core member countries. Eur. J. Polit. Econ..

[CR10] Basse T (2020). Solvency II and sovereign credit risk: additional empirical evidence and some thoughts about implications for regulators and lawmakers. Int. Rev. Law Econ..

[CR11] Basse T, Friedrich M, Kleffner A (2012). Italian government debt and sovereign credit risk: an empirical exploration and some thoughts about consequences for European insurers. Z. Gesamt. Versicherungswiss..

[CR204] Basse T, Wegener C, Kunze F (2018). Government bond yields in Germany and Spain — empirical evidence from better days. Quantitative Finance.

[CR12] Basse T, Friedrich M, Kleffner A, von der Schulenburg J-M (2014). Are interest rates too low? Empirical evidence and implications for German life insurers. Z. Gesamt. Versicherungswiss..

[CR13] Battiston, S., Jakubik, P., Monasterolo, I., Riahi, K., van Ruijven, B.: Climate risk assessment of the sovereign bond portfolio of European insurers. EIOPA financial stability report, December 2019. 69–89 (2019). https://www.eiopa.europa.eu/sites/default/files/publications/reports/eiopa_dec2019_financial-stability-report-thematic_review_climate_risk.pdf, Accessed 28 Nov 2020

[CR14] Bauer D, Maynard A (2012). Persistence-robust surplus-lag Granger causality testing. J. Econom..

[CR15] Bénassy-Quéré, A., Boot, A., Fatás, A., Fratzscher, M., Fuest, C., Giavazzi, F., Marimon, R., Martin, P., Pisani-Ferry, J., Reichlin, L., Schoenmaker, D., Teles, P., Weder die Mauro, B.: A proposal for a Covid credit line (2020). https://voxeu.org/article/proposal-covid-credit-line, Accessed 14 Dec 2020

[CR16] Benmelech E, Tzur-Ilan N (2020). The determinants of fiscal and monetary policies during the COVID-19 crisis.

[CR17] Berdin E, Gründl H (2015). The effects of a low interest rate environment on life insurers. Geneva Pap. Risk Insur..

[CR19] Bernoth K, Dany-Knedlik G, Gibert A (2020). ECB and fed monetary policy measures against the economic effects of the Coronavirus pandemic have little effect.

[CR18] Bernoth K, von Hagen J, Schuhknecht L (2012). Sovereign risk premiums in the European government bond market. J. Int. Money Finance.

[CR20] Bijlsma M, Vermeulen R (2016). Insurance companies’ trading behaviour during the European sovereign debt crisis: flight home or flight to quality?. J. Financial Stab..

[CR207] Blanchard, O., Pisani-Ferry, J.: Monetisation: Do not panic. Vox CEPR Policy Portal. https://voxeu.org/article/monetisation-do-not-panic (2020). Accessed 15 July 2020

[CR21] Boeckx, J., Deroose, M., Vincent, E.: The ECB’s monetary policy response to COVID-19. NBB Econ. Rev. (2), 37–52 (2020)

[CR22] Boonen TJ (2017). Solvency II solvency capital requirement for life insurance companies based on expected shortfall. Eur. Actuar. J..

[CR23] Botta A, Caverzasi E, Russo A (2020). Fighting the COVID-19 emergency and re-launching the European economy: debt monetization and recovery bonds.

[CR24] Boubaker S, Gounopoulos D, Nguyen DK, Paltalidis N (2017). Assessing the effects of unconventional monetary policy and low interest rates on pension fund risk incentives. J. Bank. Finance.

[CR25] Boudoukh J, Whitelaw RF (1993). Liquidity as a choice variable: a lesson from the Japanese government bond market. Rev. Financ. Stud..

[CR26] Boysen-Hogrefe J (2017). Risk assessment on Euro area government bond markets—the role of governance. J. Int. Money Finance.

[CR27] Braun A, Schmeiser H, Schreiber F (2017). Portfolio optimization under solvency II: implicit constraints imposed by the market risk standard formula. J. Risk Insur..

[CR28] Braun A, Schmeiser H, Schreiber F (2018). Return on risk-adjusted capital under solvency II: implications for the asset management of insurance. Geneva Pap. Risk Insur. Pract..

[CR29] Brunnermeier MK (2021). De-and inflationary traps: strengthening ECB’s second pillar to avoid fiscal and financial dominance.

[CR30] Burriel P, Galesi A (2018). Uncovering the heterogeneous effects of ECB unconventional monetary policies across Euro area countries. Eur. Econ. Rev..

[CR31] Chaumont, G.: Sovereign debt, default risk and the liquidity of government bonds. University of Rochester (2020). 10.2139/ssrn.3714870

[CR32] Chodorow-Reich G, Ghent A, Haddad V (2021). Asset insulators. Rev Financ Stud.

[CR33] Claeys, G.: The European Central Bank in the COVID-19 crisis: whatever it takes, within its mandate. European Parliament Monetary Dialogue Papers (2020)

[CR34] Conner R (2016). Bubble or overblown: ambiguous signals in the subprime auto lending market. J. Struct. Finance.

[CR35] Croce DR, Stewart F, Yermo J (2011). Promoting longer-term investment by institutional investors: selected issues and policies. OECD J. Financial Mark. Trends.

[CR37] Dincecco M (2009). Political regimes and sovereign credit risk in Europe, 1750–1913. Eur. Rev. Econ. Hist..

[CR38] Doff R (2008). A critical analysis of the solvency II proposals. Geneva Pap. Risk Insur. Pract..

[CR39] Doff R (2016). The final solvency II framework: will it be effective?. Geneva Pap. Risk Insur..

[CR40] Domanski D, Shin HS, Sushko V (2017). The hunt for duration: not waving but drowning?. IMF Econ. Rev..

[CR41] Düll R, König F, Ohls J (2017). On the exposure of insurance companies to sovereign risk—portfolio investments and market forces. J. Financial Stab..

[CR45] ECB: Decisions taken by the Governing Council of the ECB (in addition to decisions setting interest rates) (2020c). https://www.ecb.europa.eu/press/govcdec/otherdec/2020/html/ecb.gc200731, Accessed 14 Dec 2020

[CR42] ECB: Euro area insurers and the low interest rate environment. Financial stability review, november 2015 (2015). https://www.ecb.europa.eu/pub/pdf/fsr/financialstabilityreview201511.en.pdf, Accessed 14 July 2020

[CR48] ECB: Inflation rate (HICP) (2021a). https://sdw.ecb.europa.eu, Accessed 18 June 2021

[CR47] ECB: Monetary policy accounts: meeting of 29–30 April 2020 (2020e). https://www.ecb.europa.eu/press/accounts/2020/html/ecb.mg200522, Accessed 14 Dec 2020

[CR43] ECB: Pandemic emergency purchase programme (PEPP) (2020a). https://www.ecb.europa.eu/mopo/implement/pepp/html/index.en.html, Accessed 14 Dec 2020

[CR49] ECB: Pandemic emergency purchase programme (PEPP) (2021b). https://www.ecb.europa.eu/mopo/implement/pepp/html/index.en.html, Accessed 7 Oct 2021

[CR44] ECB: Press release: ECB announces package of temporary collateral easing measures (2020b). https://www.ecb.europa.eu/press/pr/wfs/html/index.en.html, Accessed 14 Dec 2020

[CR50] ECB: The definition of price stability (2021c). https://www.ecb.europa.eu/mopo/strategy/pricestab/html/index.en.html, Accessed 7 Oct 2021

[CR51] ECB: The ECB’s monetary policy strategy statement (2021d). https://www.ecb.europa.eu/home/search/review/html/ecb.strategyreview_monpol_strategy_statement.en.html, Accessed 7 Oct 2021

[CR46] ECB: The impact of the ECB’s monetary policy measures taken in response to the COVID-19 crisis. Economic Bulletin Boxes. 5, 37–43 (2020d). https://www.ecb.europa.eu/pub/economic-bulletin/focus/2020/html/ecb.ebbox202005_03, Accessed 14 Dec 2020

[CR52] Eichengreen B, Borner S, Grubel H (1992). Is Europe an optimum currency area?. The European Community after 1992.

[CR53] EIOPA: EEA Group quarterly balance sheet statistics (2020a). https://www.eiopa.europa.eu/tools-and-data/insurance-statistics_en, Accessed 28 Nov 2020

[CR54] EIOPA: Solo quarterly asset exposure statistics (2020b). https://www.eiopa.europa.eu/tools-and-data/insurance-statistics_en, Accessed 28 Nov 2020

[CR55] EIOPA: Solo quarterly balance sheet data (2020c). https://www.eiopa.europa.eu/tools-and-data/insurance-statistics_en, Accessed 28 Nov 2020

[CR56] Ejsing J, Lemke W (2011). The Janus-headed salvation: sovereign and bank credit risk premia during 2008–2009. Econ. Lett..

[CR57] Elderfield M (2009). Solvency II: setting the pace for regulatory change. Geneva Pap. Risk Insur. Pract..

[CR201] Eling M, Holder S (2013). Maximum Technical Interest Rates in Life Insurance in Europe and the United States: An Overview and Comparison. The Geneva Papers on Risk and Insurance — Issues and Practice.

[CR58] Eling M, Schmeiser H, Schmit JT (2007). The solvency II process: overview and critical analysis. Risk. Manag. Insur. Rev..

[CR59] European Parliament: Directive 2009/138/EC of the European parliament and of the council of 25 november 2009 on the taking-up and pursuit of the business of insurance and Reinsurance (solvency II) (recast). Official journal of the European Union (2009on). https://eur-lex.europa.eu/legal-content/EN/TXT/PDF/?uri=CELEX, Accessed 18 Oct 2020

[CR60] European Parliament: Regulation (EU) no 462/2013 of the European parliament and of the council of 21 May 2013 amending regulation (EC) no 1060/2009 on credit rating agencies (2013). https://eur-lex.europa.eu/legal-content/EN/TXT/PDF/?uri=CELEX:32013R0462&from=de, Accessed 18 Oct 2020

[CR61] Fache Rousová, L., Giuzio, M.: Insurers’ investment strategies: pro- or countercyclical?. European central bank working paper series no. 2299 (2019). https://www.ecb.europa.eu/pub/research/working-papers/html/index.en.html, Accessed 13 Dec 2020

[CR62] Filoso V, Panico C, Papagni E, Purificato F, Suárez VM (2021). Timing does matter: institutional flaws and the European debt crisis. Rev. Polit. Econ..

[CR63] Focarelli D, Marano P, Siri M (2017). Why insurance regulation is crucial for long-term investment and economic growth. Insurance regulation in the European Union. Insurance regulation in the European Union.

[CR64] Gatzert N, Wesker H (2012). A comparative assessment of Basel II/III and solvency II. Geneva Pap. Risk Insur. Pract..

[CR65] German Association of Actuaries: Hoechstrechnungszins in der Lebensversicherung (2020). https://aktuar.de/unsere-themen/lebensversicherung/hoechstrechnungszins/Seiten/default.aspx, Accessed 14 Dec 2020

[CR66] Gibson HD, Hall SG, Tavlas GS (2014). Fundamentally wrong: market pricing of sovereigns and the Greek financial crisis. J. Macroecon..

[CR67] Goldreich D, Hanke B, Nath P (2005). The price of future liquidity: time-varying liquidity in the US treasury market. Rev. Financ..

[CR70] Gormus A, Nazlioglu S, Soytas U (2018). High-yield bond and energy markets. Energy Econ..

[CR71] Granger CW (1969). Investigating causal relations by econometric models and cross-spectral methods. Econometrica.

[CR72] Grima S (2017). The ORSA requirement: insurance practitioners’ concerns.

[CR75] Grund S (2017). Restructuring government debt under local law: the Greek case and implications for investor protection in Europe. Cap. Mark. Law J..

[CR73] Gründl H, Gal J (2013). Own risk and solvency assessment within the solvency II framework and its interplay with the quantitative solvency capital requirements.

[CR74] Gründl H, Dong MI, Gal J (2017). The Evolution of Insurer Portfolio Investment Strategies for Long-term Investing. OECD J. Financial Mark. Trends..

[CR76] Gruppe M, Lange C (2014). Spain and the European sovereign debt crisis. Eur J Polit Econ.

[CR77] Gruppe M, Basse T, Friedrich M, Lange C (2017). Interest rate convergence, sovereign credit risk and the European debt crisis: a survey. J. Risk Finance.

[CR78] Gunay S (2020). Seeking causality between liquidity risk and credit risk: TED-OIS spreads and CDS indexes. Res. Int. Bus. Finance.

[CR68] Gómez-Puig M (2006). Size matters for liquidity: evidence from EMU sovereign yield spreads. Econ Lett.

[CR69] Gómez-Puig M, Sosvilla-Rivero S (2014). Causality and contagion in EMU sovereign debt markets. Int. Rev. Econ. Finance.

[CR79] Haas J, Neely CJ (2020). Central bank responses to COVID-19. Econ. Synopses.

[CR80] Hartmann, P., Borgioli, S., Kempf, A., Molitor, P., Mongelli, F.P.: Financial integration and structure in EMU during the corona crisis (2021). https://voxeu.org/article/financial-integration-and-structure-emu-during-corona-crisis, Accessed 19 June 2021

[CR81] Holder M (1999). The Euro impact on European financial markets. Manag. Finance.

[CR82] Hopt KJ (2013). Corporate governance of banks and other financial institutions after the financial crisis. J. Corp. Law Stud..

[CR208] Hutchinson, J., Mee, S.: The impact of the ECB’s monetary policy measures taken in response to the COVID-19 crisis. ECB Economic Bulletin Boxes **5**. https://www.ecb.europa.eu//pub/economic-bulletin/focus/2020/html/ecb.ebbox202005_03~12b5ff68bf.en.html (2020). Accessed 14 July 2020

[CR122] Van Hulle K (2011). Solvency II: state of play and perspectives. Z Gesamt. Versicherungswiss..

[CR83] Jankowitsch R, Mösenbacher H, Pichler S (2006). Measuring the liquidity impact on EMU government bond prices. Eur. J. Finance.

[CR84] Jareño F, Tolentino M, González MDLO, Medina MÁ (2020). Interest rate exposure of European insurers. Int. J. Econ. Bus..

[CR85] Jinjarak Y, Ahmed R, Nair-Desai S, Xin W, Aizenman J (2020). Pandemic shocks and fiscal-monetary policies in the Eurozone: COVID-19 dominance during January-June 2020.

[CR205] Kim SJ, Moshirian F, Wu E (2006). Evolution of international stock and bond market integration: Influence of the European Monetary Union. Journal of Banking and Finance.

[CR86] Klose J, Weigert B (2014). Sovereign yield spreads during the Euro crisis: fundamental factors versus redenomination risk. Int. Finance.

[CR87] Kool CJ (2000). International bond markets and the introduction of the Euro. Fed. Reserve Bank St. Louis Rev..

[CR88] Krampf A (2016). From transparency to ambiguity: the impact of the ECB’s unconventional policies on the EMU. J. Eur. Integr..

[CR89] Krishnamurthy A, Nagel S, Vissing-Jorgensen A (2018). ECB policies involving government bond purchases: impact and channels. Rev. Financ..

[CR90] Kunze F, Basse T, Gonzalez MR, Vornholz G (2020). Forward-looking financial risk management and the housing market in the United Kingdom: is there a role for sentiment indicators?. J. Risk Finance.

[CR91] Lapavitsas C (2018). The Redenomination risk of exiting the Eurozone: an estimation based on the Greek case. Eur. Law J..

[CR92] Lempérière Y, Deremble C, Nguyen TT, Seager P, Potters M, Bouchaud JP (2017). Risk premia: asymmetric tail risks and excess returns. Quant. Finance..

[CR93] Liebwein P (2006). Risk models for capital adequacy: applications in the context of solvency II and beyond. Geneva Pap. Risk Insur. Pract..

[CR94] Ludwig A (2014). Credit risk-free sovereign bonds under solvency II: a cointegration analysis with consistently estimated structural breaks. Appl. Financial Econ..

[CR95] Lund J (1999). A model for studying the effect of EMU on European yield curves. Eur. Finance Rev..

[CR96] Maltritz D (2012). Determinants of sovereign yield spreads in the Eurozone: a Bayesian approach. J Int Money Finance.

[CR97] Maltritz D, Molchanov A (2014). Country credit risk determinants with model uncertainty. Int. Rev. Econ. Finance.

[CR203] Meier S, Rodriguez Gonzalez M, Kunze F (2020). The global financial crisis, the EMU sovereign debt crisis and international financial regulation: lessons from a systematic literature review. International Review of Law and Economics.

[CR98] Möhlmann A (2021). Interest rate risk of life insurers: evidence from accounting data. Financ Manage.

[CR99] Monkiewicz J (2013). Dialectics of the current regulatory and supervisory developments in insurance. Geneva Pap. Risk Insur. Pract..

[CR100] Nazlioglu S, Gormus A, Soytas U (2019). Oil prices and monetary policy in emerging markets: structural shifts in causal linkages. Emerg. Mark. Finance Trade.

[CR101] Niedrig T (2015). Optimal asset allocation for interconnected life insurers in the low interest rate environment under solvency regulation. J. Insur. Issues.

[CR102] Officer MS (2007). The price of corporate liquidity: acquisition discounts for unlisted targets. J financ econ.

[CR103] Oliveira L, Curto JD, Nunes JP (2012). The determinants of sovereign credit spread changes in the Euro-zone. J. Int. Financial Mark. Institutions Money.

[CR104] Oxley L (1993). Cointegration, causality and export-led growth in Portugal, 1865–1985. Econ. Lett..

[CR105] Paloviita M, Haavio M, Jalasjoki P, Kilponen J (2021). What does “below, but close to, 2 percent” mean? Assessing the ECB’s reaction function with real-time data. Int. J. Cent. Bank..

[CR106] Paltalidis N, Gounopoulos D, Kizys R, Koutelidakis Y (2015). Transmission channels of systemic risk and contagion in the European financial network. J. Bank. Finance.

[CR107] Phillips PC, Perron P (1988). Testing for a unit root in time series regression. Biometrika.

[CR108] Phillips PC, Shi S (2019). Detecting financial collapse and ballooning sovereign risk. Oxf. Bull. Econ. Stat..

[CR109] Pollard P (2003). A look inside two central banks: the European central bank and the federal reserve. Fed. Reserve Bank St. Louis Rev..

[CR110] Quaglia L (2011). The Politics of Insurance Regulation and Supervision Reform in the European Union. Comp. Eur. Polit..

[CR111] Rae RA, Barrett A, Brooks D, Chotai MA, Pelkiewicz AJ, Wang C (2018). A review of solvency II: has it met its objectives?. Br. Actuar. J..

[CR112] Rodriguez Gonzalez M, Basse T, Tholl J (2019). Interest rate differentials and monetary policy in the European monetary union: the case of 10 and 30 year bonds. ZVersWiss.

[CR113] Rodriguez-Gonzalez M, Kunze F, Schwarzbach C, Dieng C (2017). Asset liability management and the Euro crisis: sovereign credit risk as a challenge for the German life insurance industry. J. Risk Finance.

[CR114] Sensoy A, Nguyen DK, Rostom A, Hacihasanoglu E (2019). Dynamic integration and network structure of the EMU sovereign bond markets. Ann. Oper. Res..

[CR115] Sibbertsen P, Wegener C, Basse T (2014). Testing for a break in the persistence in yield spreads of EMU government bonds. J. Bank. Finance.

[CR209] Sims CA (1980). Macroeconomics and reality. Econometrica.

[CR116] Smith MJ-H (2010). Solvency II: the ambitious modernization of the prudential regulation of insurers and reinsurers across the European Union (EU). Connect. Insur. Law Journal..

[CR117] Statista Research Department: Laufende Verzinsung der Lebensversicherer in Deutschland von 2000 bis 2019 (2020). https://de.statista.com/statistik/daten/studie/168461/umfrage/ueberschussbeteiligung-der-lebensversicherer-seit-1995/, Accessed 15 Dec 2020

[CR118] Tholl J, Schwarzbach C, Pittalis S, von Mettenheim HJ (2020). Bank funding and the recent political development in Italy: what about redenomination risk?. Int Rev Law Econ.

[CR119] Thornton J (1996). Cointegration, causality and export-led growth in Mexico, 1895–1992. Econ Lett.

[CR120] Toda HY, Yamamoto T (1995). Statistical inference in vector autoregressions with possibly integrated processes. J Econom.

[CR121] Valiante D (2011). The Eurozone debt crisis: from its origins to a way forward.

[CR202] Van Riet A (2017). The ECB’s Fight against Low Inflation: On the Effects of Ultra-Low Interest Rates. International Journal of Financial Studies.

[CR123] Von Saldern, N.: Government bonds: treatment of risk under solvency II. BaFin (2016). https://www.bafin.de/SharedDocs/Veroeffentlichungen/EN/Fachartikel/2016/fa_bj_1607_staatsanleihen_en.html, Accessed 7 Jan 2021

[CR36] De Vries T, De Haan J (2016). Credit ratings and bond spreads of the GIIPS. Appl. Econ. Lett..

[CR206] Wegener C, Kruse R, Basse T (2019). The walking debt crisis. Journal of Economic Behavior and Organization.

[CR124] Zabala JA, Prats MA (2020). The unconventional monetary policy of the European central bank: effectiveness and transmission analysis. World Econ..

[CR125] Zapata HO, Rambaldi AN (1997). Monte Carlo evidence on cointegration and causation. Oxf. Bull. Econ. Stat..

